# Engaging Mothers on the Growth of School-Age Children in a Rural South African Health and Demographic Site: A Qualitative Insight

**DOI:** 10.3390/healthcare9020225

**Published:** 2021-02-18

**Authors:** Perpetua Modjadji

**Affiliations:** Department of Public Health, School of Health Care Sciences, Sefako Makgatho Health Sciences University, 1 Molotlegi Street, Ga-Rankuwa, Pretoria 0208, South Africa; Perpetua.modjadji@smu.ac.za; Tel.: +27-125-213-664

**Keywords:** school-age children, growth, mothers, qualitative research, rural context, South Africa

## Abstract

A qualitative study was conducted to explore mothers’ insights on the growth of school-age children in a rural Health and Demographic site of Limpopo Province, in South Africa. The participants were selected using purposive sampling. Data were collected from seven focus group discussions, which were audio-taped and transcribed verbatim. NVivo10 was used to analyse interview transcripts, following qualitative thematic analysis. Fifty-four mothers aged between 27 and 52 years were interviewed. Unfavourable sociodemographic status with poor living conditions of mothers were observed, particularly in terms of unemployment, minimal tertiary education, and rural locality. The perceptions of mothers on child growth linked growth of their children to various factors such as poverty and socioeconomic status, genetic/family heredity, and household environment. Mothers further related child growth to purchasing power and decisions regarding types of food, food unavailability, affordability issues, feeding beliefs and practices; and child food preferences, school feeding schemes, and maternal and societal cultural beliefs and practices. Despite their concerns, mothers perceived that their children were growing well, but differently. It is worth noting that the views of mothers on child growth were up to their aptitude level and might have been restricted due to their level of education and rural locality. Hence, there is a need for novel information, education, and communication strategies to effectively reach mothers, especially in rural areas, regarding the importance of identifying children with growth failure and its prevention. Mothers should be able to identify when a child is affected by growth failure and to seek healthcare, in order to prevent children from progressing to severe forms. This study informs on the timing of nutritional interventions for children and context-specific health promotion and health education programs to improve the knowledge of mothers on child growth.

## 1. Introduction

Child growth is one of the most important health indicators from early infancy until puberty and remains one of the major public health concerns in low- and middle-income countries (LMICs) [1,2,3,4]. Child growth occurs through a complex, organized process characterized by predictable developmental stages and events [5]. A child’s growth is normal when it is within the expected growth trajectory in terms of age, height, and weight, a process that demands optimal nutrition [3,6,7]. The rate of growth differs and depends on the age of the child. Child growth is higher in the first few months after birth and the period after puberty, called a growth spurt, with more moderate growth happening between growth spurts [2,8]. Growth is the result of a complex network of many regulatory factors with varying interactions, such as nutrition, parent’s behaviours, parenting, social and cultural practices, and environment, in addition to the sociodemographic factors [9]. Normal child growth is positively associated with cognitive development, higher school achievements, lower morbidity and mortality, higher economic productivity in adulthood, and better maternal reproductive outcomes [10,11,12].

The flip side of normal child growth is growth failure, expressed as stunting, wasting, and underweight, and is a specific subset of undernutrition characterized by insufficient height or weight against age-specific growth reference standards [13,14]. Within LMICs, there is considerable variation in stunting between regions, within countries, and between households [15]. In Sub-Saharan Africa, cross-sectional studies have demonstrated an increasing prevalence of stunted growth with age, although it is commonly believed that stunting occurs mainly in young children [16,17,18,19]. In South Africa, childhood undernutrition, including school children, continues to be a public health concern [20,21]. Poverty, poor nutrition, and deprived social factors hinder children from attaining their full developmental potential [9]. In terms of the sociocultural context, the differences in household resources, community factors, and healthcare between rich and poor children affect their environments and the practices of their families and communities, ultimately affecting the children’s nutrition, health, and growth development [22]. Children with stunted growth have reduced learning abilities in school and poor scholastic achievement, are more likely to repeat grades in school or drop out, and may be at higher risk of not completing primary or secondary education [23,24,25].

It is documented that children come into middle childhood having already accrued significant deficits in growth status as assessed by stunting and underweight [18,26,27], and they experience a slower phase of linear growth during middle childhood [28]. During middle childhood, the processes of catch up growth, stable growth, or continuous growth faltering could take place among children [29]. However, it has been reported that during middle childhood, children do not catch up or even remain stable, but instead experience progression of stunted growth [29]. A catch-up growth occurs for height velocity above the statistical limits of normality for age and gender when the gap between a child’s height-for-age z scores and that of international reference standards closes [30]. Children may grow at approximately the same velocity as that of children in better environments, such that they complete middle childhood with only the deficits accrued during the first few years of life (i.e., stable growth). On the other hand, children may continue to falter, growing at a slower velocity than children in better environments, such that deficits during middle childhood are added to those accrued during the first few years of life [29,30].

Several frameworks have been developed over the years to understand the determinants of child growth, development, health, and survival [31,32,33]. A combination of the UNICEF conceptual framework for malnutrition of children [34] and the Bronfenbrenner’s social ecological model for child growth and development [35] was used to conceptualize the current study. The UNICEF [34] framework explains that the three levels of causes of child malnutrition are, first, the immediate causes, which entail inadequate dietary intake and disease, then follow the underlying causes, which are insufficient access to food, inadequate maternal and child-care practices, poor water/sanitation, and inadequate health services. The last level are the basic causes include widespread poverty, increasing inequality, and suboptimal public services, rooted in an inequitable economic system. On the other hand, Bronfenbrenner [36] has indicated that there are different contexts in which human development takes place. Family, as an immediate context, has a crucial impact on child development [37]. Parents or primary caregivers care for the child and provide him/her with food and protection against threats [38,39]. The influence of parents or primary caregivers is especially important not only in the early stages, but also even in the adolescence [40]. Another important immediate context for a child’s development is school [41,42]. Additionally, there are other more distal contexts, such as the family’s social network and community [36,43], and the broader context of ethnicity [44,45], including, for example, social values [46,47].

Until recently, there was a paucity of data on the growth status of school-aged children in the developing world, with most work focusing on children under five. Yet, growth faltering is of great public health significance from infancy to puberty, as attained height has important implications for adult work capacity and reproductive outcomes [48,49]. Parents’ ability to identify when a child is undernourished and their decision-making around seeking healthcare have been raised as major concerns pertaining to child growth, and social, cultural, and economic contexts have been implicated [50]. Culturally, mothers play an important role in the growth of their children [6]. With a paucity of qualitative research on child growth, this paper explored the insights of mothers on growth of their school-age children in a rural health and demographic site of Limpopo Province in South Africa. It is important to understand the dynamics of linear growth during middle childhood, as the timing of nutritional interventions depends on identifying periods of faltering [29].

## 2. Materials and Methods

### 2.1. Study Design, Setting and Population

The current study was nested within a larger study that investigated growth patterns (i.e., malnutrition) and sociocultural beliefs and practices in Dikgale, Limpopo Province: A mixed method study of primary school children and their mothers (Doctoral thesis) [51]. This paper reports on a qualitative strand, which explored the mothers’ influence and views on growth and nutrition of school-age children in the study site. The study adhered to the Consolidated Criteria for Reporting Qualitative Research (COREQ) [52,53]. The importance of using various approaches to analyse the drivers of growth failure, ranging from household surveys to qualitative approaches and mixed methods research has been recognized [54], but there is a gap in literature regarding qualitative insights of mothers on the growth of school-age children. Hence, use of a qualitative approach as one of the methods in a larger study enabled us to produce an in-depth understanding of mothers’ opinions on growth of school-age children from a rural context [55]. Papers on a quantitative strand from a larger study have been published [56,57,58].

The study was conducted in a rural Dikgale Health and Demographic Surveillance System Site (Dikgale HDSS site or DHDSSS) described in detail by Alberts et al. [59]. Briefly, Dikgale HDSS site is situated approximately 40 km north-east of Polokwane, the capital city of the Limpopo Province, in South Africa. The area comprises of communities clustered in 16 villages with a population of approximately 36,000 and has poor infrastructure [59]. There are 19 public primary schools under the Dimamo Education Circuit in the villages forming part of the Dikgale HDSS site [39].

Fifty-four mothers of school-age children who participated first in the quantitative strand participated in seven focus group discussions (FGDs). Mothers of school-age children were recruited telephonically, using the contact details they provided during a quantitative component, and interviewed about the growth of their school-age children. This is because mothers spend considerably more time with their children than fathers do, hence, in most cases, they are held more accountable for the health, nutrition, growth, and development of their children [60,61,62].

### 2.2. Data Collection, Tools, and Procedure

A semistructured interview guide (see Supplementary Materials) was self-developed and informed by the conceptual framework, which combined the UNICEF framework of child malnutrition and the Bronfenbrenner’s socioecological model, as explained earlier, to address the objectives of the larger study. The discussions were focused on the mothers’ insight on growth and nutrition of school-age children. The interview guide was developed in English and translated into a local language (i.e., Sepedi). The interview schedule consisted of open-ended questions covering topics such as understanding about growth of school-age children and opinions on how food and feeding practices affect child growth. Further questions were on the adequacy of food on a daily basis at home or at school, opinions on types of foods, the beliefs that influence which foods to eat, and the views on the way culture prescribes the kinds of foods for children. The tool was pretested, revised, and translated back to English. The guide consisted of seven questions, which were modified as the data collection proceeded. The enquiry exchange between the researcher and the participants was well-managed. In addition, follow-up questions and predefined probes were asked in response to the responses given by the participants. Each FGD consisted of 6–12 members, took approximately 60–90 min, and was recorded with the consent of the participants, which was obtained before the interviews took place. A facilitator moderated the discussion while a notetaker took handwritten notes. Data saturation was reached when there was enough information obtained from the FDGs to replicate the study, when the ability to obtain additional new information was attained, and when further coding was no longer feasible, as we frequently obtained repeat/identical information The FGDs were conducted in school classrooms or quiet places allocated by the school. In case the school had no free classroom, FGDs were conducted at the kraal of the local chief, where there was a community building with quiet rooms. In addition, sociodemographic variables included age, marital status, education level, unemployment status, income, access to social grants, and household information.

### 2.3. Data Management and Analysis

An experienced transcriptionist transcribed verbatim all the interviews from the audio files that used the language of the participants to best represent the dynamic nature of the living conversation. Transcripts were translated into English and reviewed by the researcher to ensure their accuracy and that no meaning was lost between the transcription and the translation. Thematic analysis was used to analyse data [63]. First, transcripts were read by the researcher to familiarize and immerse herself with the data to the extent of being familiar with the depth and breadth of the content. Second, the initial codes were generated from the data using manual coding of a few transcripts. Then followed the development of the codebook, where the themes were reviewed, refined, and named. The themes were given definitions that determined the essence of what each theme was about and determined what aspect of the data each theme captured. Once the codebook had been developed, consensus about the themes was reached between the researcher and the supervisor. Then, the transcripts were imported into NVivo QSR version 11 (QSR International, Melbourne, Australia), for storage and organization of files such as interview transcripts, field notes, and interview summaries. The findings were presented in themes and quotations that reflected mothers’ views regarding the growth and nutrition of school-age children. To ensure rigor, data was analysed by the author and a qualitative expert to reduce the effect of researcher bias, and to ensure that the interpretations were free from bias and the conclusions were credible. The study employed investigator triangulation, whereby the FGDs were facilitated by the researcher, the supervisor, and the moderator—who is an experienced qualitative researcher. Further strategies were used to ensure that the results were credible, and the researcher maintained a reflective attitude throughout the data collection and analysis process [64].

### 2.4. Ethical Considerations

This study received ethical clearance from Sefako Makgatho Health Sciences University Research and Ethics Committee (SMUREC) (SMUREC/H/161/2016: PG). Participation was voluntary and the participants provided written informed consent. The researcher used pseudonyms to report the data and maintained confidentiality at all times.

## 3. Findings

### 3.1. Sociodemographic Characteristics of the Participants

As shown in Table 1, the ages of the mothers (*n* = 54) ranged from 27 to 52 years (mean age 37 ± 7 years). Half of the mothers (57.4%) were single, 83.3% were unemployed, and 90.7% depended on child social grants. Most mothers lived in larger households of 5–9 members (55.5%). In almost half of the households (46.3%), monthly income was less than $60.66, and income was above $116.02 in only 3.7% of households. Poor sanitation (i.e., use of pit toilets (96.3%)) was reported. Mothers had homogenous characteristics in terms of their sociodemographic status, as indicated in Table 1. In addition, the results of quantitative strand in a larger study showed that 22% of schoolchildren were stunted, 27% were underweight, and 25% were thin, while only 4% were overweight and 1% were obese [56,57]. On the other hand, mothers were overweight (27%) and obese (42%), with only few being underweight (2%) [58] (results not shown).

### 3.2. Emergent Themes

Ten main themes emerged from the analyses of the FGDs. The main themes were (1) perceptions on child growth, (2) food unavailability, (3) food affordability, (4) feeding beliefs and practices, (5) decision to purchase foods, (6) child food preferences, (7) perceptions on school feeding schemes, (8) food knowledge (9) cultural beliefs and practices during pregnancy, and (10) societal cultural beliefs and practices. This paper focusses on the first 7 (seven) themes. The themes are described in Table 2.

#### 3.2.1. Theme 1: Perceptions on Child Growth

When asked about their understanding on child growth, mothers seem to believe that their children are growing well, but differently. They described various factors, which they considered important to affect the growth of their children. Based on their narratives, such factors included socioeconomic status and poverty, genetic/family heredity, food consumption, maternal feeding practices, and household environments.

***Socioeconomic status and poverty***. Mothers linked poverty to child growth. One mother noted that children grow better when they come from rich families than children from poor families:

“There is a difference between a child from a poor family and a child from a rich family. In a poor family children grow up under difficult circumstances whereas in a rich family they grow up under good circumstances.”

***Genetic/family heredity***. Most mothers cited genetics as a factor when asked about their understanding on child growth. They noted that, as a child ages, his/her growth is affected by the stature of parents/family:

“My belief is that child’s growth is determined by heredity from the family. For an example, my husband is skinny and my first-born child is also skinny, but my second child is fat, she is bigger than the sister is…, the sister is skinny and tall. I am saying this because sometimes children take the genes of the mother or the father.”

***Food consumption***. Mothers described intake of a variety of foods as an indicator of a healthy child growth. As one mother said: *“I think the growth of children can be affected by the kind of food we give them every day.”.* One mother went further on to explain that meat might help children to grow well compared to when they eat pap and water: *“They must also eat meat…, sometimes you find children eating pap and water…, those children will not grow up well”*.

***Maternal feeding practices***. Mothers indicated that child growth is affected by proper feeding practices:

“You will find that a child who is not growing well they do not have anything to eat in the morning in their households, when they wake up in the morning they eat left over pap while the child who grows up well eat cornflakes in the morning.”

***Household environment***. Mothers recognized that, as children age, their growth coincides with the environment of their upbringing. However, mothers narrated the household environment in terms of parents relating to one another and the treatment they give to children: *“Another contributing factor in the child’s growth is the situation at home. If parents are always fighting, that affects children negatively”*. Another comment made was that *“As the child goes out of their home we must be able to see how they are treating them in their households the way the child looks it will show how they are treated in their homes that will show if they grow up well or not”*.

#### 3.2.2. Theme 2: Food Unavailability

Some mothers expressed substantial concerns over food not always being available or enough in the household, with the understanding that food is important for child growth. The most cited reasons leading to food not being available in households were unemployment and the fact that most mothers depended on social child grant money: *“I think the reason food is not enough is because people are unemployed and the household income is lesser than the needs of the family”*. Furthermore,

*“… food is not enough because the money we get is too little and things are expensive. Social grant money is not enough to buy things that we want, we buy mealie meal, sugar and flour then the money will be finished. Then for other things to buy there will be no more money to buy them, things are expensive and they need money and that money is never enough as we speak with you now there is no food in our households.”*

#### 3.2.3. Theme 3: Food Affordability

In addition to food unavailability, mothers narrated in a hypothetical manner that having enough food in their households would entail having the basic foods such as mealie meal flour, sugar tea, in addition to fruits and vegetables, which they have only when they can afford to buy those foods. Some statements made are as follows: *“When we say food is enough in the household we talk about things like mealie meal, sugar, tea bags, and flour. If those things can be bought then I think days can move well”*.

#### 3.2.4. Theme 4: Feeding Practices and Beliefs

Mothers indicated the importance of feeding children in relation to them growing well. They cited the feeding beliefs and practices, which included feeding patterns such as early and childhood feeding, provision of lunchboxes for school, as well as the types of foods.

***Early feeding***. Mothers referred to how they handled early feeding in terms of child growth. One mother mentioned the importance of breastfeeding and that a child grows and develops well when breastfed, but further indicated the health challenges some mothers face in terms of breastfeeding and thus resort to bottle-feeding:

“It is important how you feed the child. For example, when a child is breastfed the child grows and develops well. Some mothers have health problems when it comes to breastfeeding and the child ends up feeding on bottle milk..., but breast milk has more benefits.”

***Breakfast***. When explaining how they feed their children, mothers narrated the significance of breakfast and further alluded the fact that one does not have to feed children eggs, but any kind of feeding is acceptable. One mother said,

*“Every child deserves breakfast for the child’s mental health even in class the child is able to be active. The breakfast doesn’t have to be eggs, as long as there is breakfast it will prepare the child’s mind to assist the child in thinking, so that is a very important thing to assist the child in thinking at school.”*

***Snacks***. Although mothers mentioned snacks when narrating their feeding practices and beliefs, one mother mentioned the negative effect of snacks, such as Danone yoghurt, sweets and juice on the appetite of children and she said,

*“Currently, as mothers, we feed our children with snacks and at times they make children to lose appetite on healthy food. You can breastfeed the child for six months without solid food and another mother after giving birth gives the child solid food and for some of us who have money we feed our children “Danone” yoghurt, sweets, juice and if the child feeds on those things the child loses appetite.”*

***Food choices***. Mothers also discussed the food choices based on the availability and how they believe such foods will help their children to grow well. One mother noted, *“I think we can feed them with beans, sugar beans, and fish because they have proteins and they assist in children’s mental health as they grow up, also milk, vegetables, fruits, mealie rice”*.

One mother mentioned that they plant vegetables, which enables them to cook a variety of meals from vegetables, meat, and milk on different days. She said, *“In the areas where we are staying most people have planted vegetables, we can cook different dishes on different days, today I can prepare vegetables, tomorrow meat and the next day milk, and the other day eggs, I need to change the menu and prepare healthy and clean food”*.

***Lunch boxes***. Some mothers indicated that their children carry lunchboxes to school, despite food being offered at schools. Apparently, one reason why mothers indicated a preference to prepare lunchboxes for their children is because some foods are not cooked well at schools:

“On the days when eat samp at school, my child takes a lunch box with. If I cook samp at home my child eats it, the challenge is that at school when they cook it they do not cook it well enough and it causes diarrhoea. So, I prefer to prepare lunch box for my child instead.”

#### 3.2.5. Theme 5: Decision to Purchase Foods

When asked about who decides on purchasing foods in their households, most of the mothers described their buying practices in terms of what informs the food they purchase, in addition to them taking the decision, as mothers, based on their objective:

“I think the main thing is to start by buying those things that are necessary in the house you start buying the needs first and other things will follow after … I think of buying mealie-meal, meat, toiletry, salt, shoe polish, hair products… We just buy mealie meal and meat …mealie meal, washing soap.”

In addition to mealie meal and meat, mothers reported to prioritize on foods such as rice, eggs, milk: “… *For example, in the previous month, I bought rice and it did not last for long so I bought it again but I did not buy sugar because it was still available and I just bought something else like eggs and milk*.”.

#### 3.2.6. Theme 6: Child Food Preference

Mothers reported that child eating behaviours such as food preference also influences child growth. Since children in these communities are provided food at school through the feeding scheme program, mothers reported on the food children like and dislike both at home and at school. One mother noted:

“But our children do not want to eat morogo [green leafy vegetables commonly grown in most gardens]. Right now, if you can serve them with morogo they will put it aside and eat pap with relish instead. If you cook cabbage, they will tell you that they are not hungry, even if they are hungry. For an example we have planted vegetables in our gardens and our children will tell you that they do not want them they want meat.”

One mother was able to explain child food preference in relation to some foods, such as soup, which is cooked regularly at their schools. In addition, it was made clear that children choose foods because some foods at school make them sick. She said, *“Is just that children are very choosy they do not like soup most of them I know because I once worked in a school I’ve seen them they do not like it because some of them develop rash when they eat it or diarrhoea”*.

#### 3.2.7. Theme 7: Perception of Mother about School Feeding Program

Data showed that most of the households depended on a social child grant and had inadequate food to feed their children. As a result, the FGDs revealed that most mothers perceived the food at school to be better than the food at home. Mothers narrated a variety of foods such as samp, beans, pap, and milk, that are available at schools compared to their households:

“In my opinion, the food that they give these children at school is good food and better than the food they get at their households. They are able to eat samp, beans, pap, and milk and they get fruits. At home, they do not get those things they only get them in school.”

While most of the mothers perceived that foods received at school is adequate, a few felt that the food was inadequate at schools. One mother said, *“I think the food is not enough, if the food was enough, our children wouldn’t rush to the pots the first thing when they come back from school”*.

Finally, some mothers had suggestions as to how the feeding scheme at schools could be improved to meet the needs of the children. This was explained in relation to time of feeding, rather than the menu. One mother noted:

“I am suggesting that if the teachers can shift the time of serving meals to 11:30 this will at least carry the child until school is out, and it will eliminate this thing that when the child gets home the first the child wants is food because children are different.”

## 4. Discussion

This study aimed to explore the insights of mothers on child growth in a rural health and demographic site. To my knowledge, there is a paucity of data with regard to primary school children growth status in rural settings of South Africa. Hence, comparisons of the findings of the current study with other findings is limited. Generally, mothers are the caregivers of their children as they grow, so they are able to express their viewpoints on child growth. The current study revealed that child growth was affected by factors such as genetic heredity, poverty and socioeconomic status, and household environment. Furthermore, mothers related child growth to purchasing power and food decision-making, food unavailability and affordability issues, feeding beliefs and practices, and food knowledge, as well as child food preferences, school feeding schemes, and maternal and societal cultural beliefs and practices.

Growth failure arises when poverty becomes a permanent condition, leading to cumulatively inadequate food intake, and poor health conditions [65]. Mothers in this study were single, unemployed, lived in larger households, were dependent on child social grants, and survived with a monthly income of less than $316.69. In South Africa, large numbers of households are insecure regarding food, more than half of the population live in poverty, and one third live in extreme poverty [66,67,68]. Similar living conditions characterised by household poverty have been reported in the Limpopo Province [69], where the current study was conducted. Sociodemographic characteristics have been linked to both dietary quality and feeding practices [70,71]. 

Mothers in this study indicated food unavailability due to households lacking spousal support and the power to purchase food. Thus, they could only resort to food they could afford to purchase, based on their choices as well as communication with their children to make decisions on which foods to buy. Researchers have reported that, on the one hand, a low ability to purchase food gives rise to stunting, underweight, and thinness [72]; meanwhile, other studies have reported that mothers’ employment provides financial power and independency leading to better child care and, therefore, better heights and weights of their children [6,73].

Tertiary education was minimal among the respondent mothers. Low literacy, which is independently associated with growth faltering in children, may also limit mothers’ abilities to appropriately understand the practical details of child growth and monitoring [74,75,76]. Literature documents that children from the poorest households and under the care of mothers with low education are more likely to be stunted, this could be mediated by poor knowledge of adequate health and nutritional behaviours or limited access to and use of health services [77]. However, the views of mothers on child growth were up to their aptitude level, considering their level of education and rural locality. On the other hand, lack of awareness of proper feeding practices among mothers makes maternal engagement with growth monitoring more difficult [78]. Our findings suggest that mothers lacked appropriate knowledge regarding child growth; hence, the group may be particularly vulnerable and may benefit from programs aimed at increasing knowledge on growth awareness. In addition, promotion of strategies to address some of the information gaps in the recognition of linear growth failure and its consequences among these mothers with lower literacy may be important to improve linear growth outcomes [79].

A continued experience of growth faltering during middle childhood indicates that children have insufficient intake of micronutrients, macronutrients, or both to meet the demands of normal growth [29]. Micronutrient deficiencies in South African children are directly linked to the quality of food made available to them. Child growth is disrupted when the value and amount of food consumed is poor, compromising the quantity of consumption, quality, and diversity. Food was one factor that mothers perceived as affecting child growth. Children’s growth is multifactorial, with nutrition being an important factor [80]. Narratives of mothers revealed that child food preferences affect how children grow. Mothers further explained that their children are choosy when it comes to some foods and raised concerns that some of the foods their children prefer were not good for them. Food preferences change throughout life, under the influence of biological, social, and environmental factors [81]. These preferences are key determinants of food choices and, therefore, diet quality [82,83]. Dietary quality is influenced by practices such as eating breakfast [84], family meals [85], and fast-food consumption [86].

To date, food fortification programs have been introduced to address micronutrient deficiency. Nonetheless, these programs have not significantly improved dietary diversity or macronutrient intake. Furthermore, the South African government has implemented three policy initiatives to address the underlying causes of insufficient nutrient intake. These initiatives include the provision of Child Social Grants (CSG), the Integrated Food Security and Nutrition Programme (IFSNP) and the National School Nutrition Programme (NSNP) [87]. In particular, perceptions on the NSNP indicated contrary feelings of mothers, since some expressed satisfaction with the food children receive at school while others claimed that the food at schools was insufficient to meet children’s growth needs. The Healthy Active Kids South Africa (HAKSA) has suggested that the NSNP is not meeting its objectives of providing optimal nutritious meals [88]. Hence, stunting continues to affect a proportion of South African children [87]. Association of dietary diversity with stunting and underweight has been reported among children in developing countries [89,90], including South Africa [91].

Other factors such as genetics and infection can alter growth at any age, from the birth of the child until growth terminates [92]. Genetic heredity was mentioned as a factor influencing child growth in the current study. Although normal growth of a child likely indicates good general health and well-being, there is great variability in the heights and weights that are within the range of normal [93]. Abnormal slow growth may indicate a pathological process, and height is a genetically determined parameter, affected by the interactions of hundreds of genes [93]. Genetics plays a vital role in determining the rate of growth [94]. Some genetic disorders cause growth alterations through genetic defects in the growth hormone such as insulin-like growth factor-I [95]. While mothers in this study perceived several factors, including genetic hereditary, were linked to child growth, they did not consider their nutritional status as a possible factor. Overnutrition among mothers from this current study was earlier reported in the quantitative strand, where their children were undernourished and were stunted [56,57,58]. Households where a child is stunted and the mother is overweight have been documented in several countries, including in Africa [96].

Although mothers expressed greater concern about children’s growth status in this study, similar to other findings [78], they still believed that their children were growing well, despite the high prevalence of stunting, underweight, and thinness among their children reported earlier from the same study [56,57]. Hossain et al. [78] reported that linear growth interpretation among caregivers was determined more by community norms than by guidance from nutrition programming or the health system [78]. This suggests that mothers in this study might be clueless about the potential adverse health consequences associated with growth failure and that could sustain detrimental consequences as children advance through stages of life. There is a lack of understanding of the ways in which caregivers recognize and respond to children’s linear growth, especially in settings where poor height attainment may not be perceived as abnormal [97]. In settings with a high prevalence of stunting, caregivers may fail to recognize linear growth failure in children [98].

## 5. Limitations

There are several limitations to this analysis. A broader interview guide was developed to elicit responses that may be applied elsewhere; however, the rural context of the study may not translate to other settings. The study design enabled the identification of the main themes by the participant group but did not allow expression of the frequency of these themes or to rank them in order according to their level of importance. All emergent themes of the study are considered important regarding child growth. Although most mothers had education level of below or up to grade 12, this study was able to reveal and understand the insights of mothers on the growth of school-age children at their aptitude level, and from a rural context. FGD was considered an ideal method in the current study to elicit mothers’ understandings, opinions, and views on growth of school-age children, however, use of FGD might have allowed the tendency for certain types of socially acceptable opinions to emerge, and for certain types of participants to dominate the discussion, among other limitations.

## 6. Conclusions

The present study suggests that mothers have minimal insight regarding child growth, to some extent. It seems mothers relied on their personal instincts to explain child growth. Despite their concerns, mothers perceived that their children were growing well, but differently, due to various factors such as poverty and socioeconomic status, genetics, and household environment. Furthermore, mothers related child growth to purchasing power and food decisions, food unavailability and affordability issues, feeding beliefs and practices, as well as child food preferences and school feeding schemes. It remains important for mothers to be able to identify when their children are affected by growth failure (i.e., child undernutrition) and to seek healthcare in order to prevent children from progressing to severe forms. Monitoring a child’s growth in the early stages through healthcare assists in detecting abnormalities affecting the child’s quality of life and facilitates early intervention. Minimal tertiary education and the context of rural locality might have contributed to some misconceptions mothers in our sample had about child growth and limited their insight. Hence, there is a need for novel information, education, and communication strategies to effectively reach mothers, especially in the rural settings, regarding the importance of identifying children with growth failure and its prevention. In addition, making mothers aware of the importance of growth failure, its early signs, and its long-term impacts on health and human capacity are critical for effective program development and delivery.

## Figures and Tables

**Table 1:** Sociodemographic characteristics of the mothers (*n* = 54).

Variables	Categories	Frequency	Percentages
Age (years)	25–35	21	38.9
	>35	33	61.1
Marital status	Single	31	57.4
	Ever married	24	42.6
Education level	Primary school	5	9.3
	Secondary school	18	33.3
	Completed grade 12	25	46.3
	Tertiary	6	11.1
Employment status	Yes	9	16.7
	No	45	83.3
Social grant	Yes	49	90.7
	No	5	9.3
Dwelling	Brick	33	61.1
	RDP	14	25.9
	Shack	7	13
Household head	Self	19	35.2
	Spouse	21	38.8
	Parents/grandparents/relatives	14	29.5
Household income	<$60.66	25	46.3
	$60.66–$116.02	27	50
	>$116.02	2	3.7
Household size	1–4	18	33.3
	5–9	30	55.5
	≥10	6	11.1
Household adults	≤2	40	74.1
	>2	14	25.9
Household children	≤2	14	25.9
	>2	40	74.1
Water access	Yes	31	57.4
	No	23	43.6
Refrigerators for food storage	Yes	35	64.8
	No	7	13
	Sometimes	12	22.2
Type of a toilet	Pit	52	96.3
	flush	2	3.7

RDP stands for Reconstruction and Development Programme houses.

**Table 2:** Themes that emerged from the focus group discussions (FGDs).

Emerging Themes	Description
Perceptions on child growth	Responses that mention or describe “growth” and “child growing up well” or “not growing up well” with consideration of various factors or circumstances affecting child growth.
2. Food unavailability	Refers to any mention of “food not being enough” in the households. This includes the reasons behind food not being enough in the households.
3. Food affordability	Responses that mention “food is enough, or enough food” and “afford to buy”. This includes mention of foods mothers consider basic in their households
4. Feeding beliefs and practices	Responses that mention, “early and childhood feeding”, “breakfast”, “lunchboxes”, and “food choices” as mothers practices and beliefs on feeding their children.
5. Decisions to purchase foods	Any mention of what informs the food that mothers buy in their households. This include the kinds of foods mothers buy in their households.
6. Child food preferences	Responses that mention, “children are choosy” or “children do not want to eat or they eat”. This include the foods types that children prefer to eat and those they do not.
7. Perceptions on school feeding scheme	Responses that mention or describe school feeding schemes. This includes the comparison of foods being enough or not enough, at schools and at home, including suggestions on the school feeding scheme improvement.

## Data Availability

This study used qualitative transcripts, which are stored in NVivo software (confidentially).

## Supplementary Materials

The following are available online at https://www.mdpi.com/2227-9032/9/2/225/s1. Questionnaire: Engaging mothers on child growth in a rural South African Health and Demographic site: A qualitative insight.

## References

[1] Al-Sharbati M., Younan A.A., Sudani O.H. (2001). Growth pattern of primary schoolchildren in Benghazi, Libya. J. Sci. Res. Med Sci..

[2] Hosseini S.-M., Maracy M.-R., Sarrafzade S., Kelishadi R. (2014). Child weight growth trajectory and its determinants in a sample of Iranian children from birth until 2 years of age. Int. J. Prev. Med..

[3] De P., Chattopadhyay N. (2019). Effects of malnutrition on child development: Evidence from a backward district of India. Clin. Epidemiology Glob. Health.

[4] Tekle M., Tariku B., Alagaw A., Zerihun E., Wondiye H. (2019). Exploring reasons for low attendance of mothers to growth monitoring and promotion program at Loka Abaya District, Southern Ethiopia: Exploratory qualitative study. J. Nutr. Metab..

[5] Kohl H.W., Cook H.D. (2013). Physical activity and physical education: Relationship to growth, development, and health. Educating the Student Body: Taking Physical Activity and Physical Education to School.

[6] Alhaidari S.I., Al Houssien A.O., Alteraiqi M.A., Al Arafah A.M., Al Houssien R.O., Alhaidari O.I., Omair A.I. (2016). Children’s growth pattern and mothers’ education and socio-economic status in Riyadh, Saudi Arabia. J. Health Spec..

[7] El Mouzan M.I., Al Herbish A.S., Al Salloum A.A., Al Omar A.A., Qurachi M.M. (2007). Growth charts for Saudi children and adolescents. Saudi Med. J..

[8] Doyle D. Physical Growth of Infants and Children. https://wwwmerckmanualscom/professional/pediatrics/growth-and-development/physical-growth-of-infants-and-children.

[9] Pem D. (2012). Factors affecting early childhood growth and development: Golden 1000 Days. Adv. Pract. Nurs..

[10] Kowalski A.J., Georgiadis A., Behrman J.R., Crookston B.T., Fernald L.C., Stein A.D. (2018). Linear growth through 12 years is weakly but consistently associated with language and math achievement scores at age 12 years in 4 low-or middle-income countries. J. Nutr..

[11] Prendergast A.J., Humphrey J.H. (2014). The stunting syndrome in developing countries. Paediatr. Int. Child Health.

[12] Sudfeld C.R., McCoy D.C., Danaei G., Fink G., Ezzati M., Andrews K.G., Fawzi W.W. (2015). Linear growth and child development in low- and middle-income countries: A meta-analysis. Pediatrics.

[13] World Health Organization (WHO), United Nations International Children’s Emergency Fund (UNICEF) WHO Child Growth Standards and the Identification of Severe Acute Malnutrition in Infants and Children: A Joint Statement, Geneva 2009. https://wwwwhoint/nutrition/publications/severemalnutrition/9789241598163/en/.

[14] Wang Y., Chen H.J., Preedy V.R. (2012). Handbook of Anthropometry.

[15] Shaheen F., Glennie J., Lenhardt A., Roche J. (2016). Every Last Child.

[16] Lwambo N., Brooker S., Siza J.E., Bundy D., Guyatt H. (2000). Age patterns in stunting and anaemia in African schoolchildren: A cross-sectional study in Tanzania. Eur. J. Clin. Nutr..

[17] Monyeki K., Cameron N., Getz B. (2000). Growth and nutritional status of rural South African children 3–10 years old: The Ellisras growth study. Am. J. Hum. Biol..

[18] Partnership for Child Development (1998). The anthropometric status of schoolchildren in five countries in the partnership for child development. Proc. Nutr. Soc..

[19] Martorell R., Khan L.K., Schroeder D.G. (1994). Reversibility of stunting: Epidemiological findings in children from developing countries. Eur. J. Clin. Nutr..

[20] Monyeki M.A., Awotidebe A., Strydom G.L., De Ridder J.H., Mamabolo R.L., Kemper H.C. (2015). The challenges of underweight and overweight in South African children: Are we winning or losing the battle? A systematic review. Int. J. Environ. Res. Public Health.

[21] Kruger G., Pienaar A.E., Coetzee D., Kruger S.H. (2014). Prevalence of stunting, wasting and underweight in Grade 1-learners: The NW-CHILD study. Health SA Gesondheid.

[22] World Health Organization (WHO), World Bank Working Group (2001). Better Health for Poor Children: A Special Report.

[23] Council on Hemispheric Affairs (2013). The Cost of Hunger in Ethiopia: The Social and Economic Impact of Child Undernutrition in Ethiopia. Implications for the Growth and Transformation of Ethiopia.

[24] Galal O.M., Neumann C.G., Hulett J. (2005). Nutritional deficits on the education for all agenda. Food Nutr. Bull..

[25] Mesfin F., Worku A., Berhane Y. (2015). Prevalence and associated factors of stunting among primary school children in Eastern Ethiopia. Nutr. Diet. Suppl..

[26] Martorell R., Schroeder D.G., Rivera J.A., Kaplowitz H.J. (1995). Patterns of linear growth in rural Guatemalan adolescents and children. J. Nutr..

[27] Stoltzfus R.J., Albonico M., Tielsch J.M., Chwaya H.M., Savioli L. (1997). Linear growth retardation in Zanzibari school children. J. Nutr..

[28] Tanner J.M. (1990). Foetus into Man: Physical Growth from Conception to Maturity.

[29] Friedman J.F., Phillips-Howard P., Mirel L.B., Terlouw D.J., Okello N., Vulule J.M., Hawley W.A., Nahlen B.L., Ter Kuile F.O. (2005). Progression of stunting and its predictors among school-aged children in western Kenya. Eur. J. Clin. Nutr..

[30] Boersma B., Wit J.M. (1997). Catch-up growth. Endocr. Rev..

[31] Mosley W.H., Chen L.C. (1984). An analytical framework for the study of child survival in developing countries. Popul. Dev. Rev..

[32] Stewart C.P., Iannotti L., Dewey K.G., Michaelsen K.F., Onyango A.W. (2013). Contextualising complementary feeding in a broader framework for stunting prevention. Matern. Child Nutr..

[33] Ryan J., Paquette D. (2001). Bronfenbrenner’s Ecological Systems Theory.

[34] United Nations International Children’s Emergency Fund (UNICEF) (1998). Conceptual frameworks. UNICEF M&E Training Resources.

[35] Bronfenbrenner U. (1994). Ecological models for human development. International Encylopedia for Education.

[36] Bronfenbrenner U. (1986). Ecology of the family as a context for human development: Research perspectives. Dev. Psychol..

[37] Garcia O.F., Fuentes M.C., Gracia E., Desfilis E.S., Garcia F. (2020). Parenting warmth and strictness across three generations: Parenting styles and psychosocial adjustment. Int. J. Environ. Res. Public Health.

[38] Garcia O.F., Desfilis E.S., Zacares J.J., Calafat A., Garcia F. (2020). Alcohol use and abuse and motivations for drinking and non-drinking among Spanish adolescents: Do we know enough when we know parenting style?. Psychol. Health.

[39] Queiroz P., Garcia O.F., Garcia F., Zacares J.J., Camino C.P.D.S. (2020). Self and nature: Parental socialization, self-esteem, and environmental values in Spanish adolescents. Int. J. Environ. Res. Public Health.

[40] Fuentes M.C., Alarcón A., García F., Gracia E. (2015). Consumo de alcohol, tabaco, cannabis y otras drogas en la adolescencia: Efectos de la familia y el barrio [Use of alcohol, tobacco, cannabis and other drugs in adolescence: Effects of family and neighborhood]. An. Psicol..

[41] Aunola K., Stattin H., Nurmi J.-E. (2000). Parenting styles and adolescents’ achievement strategies. J. Adolesc..

[42] Eccles J.S., Midgley C., Wigfield A., Buchanan C.M., Reuman D., Flanagan C., Mac Iver D. (1993). Development during adolescence: The impact of stage-environment fit on young adolescents’ experiences in schools and in families. Am. Psychol..

[43] Steinberg L., Darling N.E., Fletcher A.C. (1995). Authoritative parenting and adolescent adjustment: An ecological journey. Examining Lives in Context: Perspectives on the Ecology of Human Development.

[44] Darling N., Steinberg L. (1993). Parenting Style as Context: An Integrative Model.

[45] Pinquart M., Kauser R. (2018). Do the associations of parenting styles with behavior problems and academic achievement vary by culture? Results from a meta-analysis. Cult. Divers. Ethn. Minor. Psychol..

[46] Gracia E., García F., Lila M. (2014). Male police officers’ law enforcement preferences in cases of intimate partner violence versus non-intimate interpersonal violence: Do sexist attitudes and empathy matter?. Crim. Justice Behav..

[47] Lila M., Gracia E., García F. (2013). Ambivalent sexism, empathy and law enforcement attitudes towards partner violence against women among male police officers. Psychol. Crime Law.

[48] Norgan N.G. (2000). Long-term physiological and economic consequences of growth retardation in children and adolescents. Proc. Nutr. Soc..

[49] Martorell R., Delgado H.L., Valverde V., Klein R.E. (1981). Maternal stature, fertility and infant mortality. Hum. Biol..

[50] Kleinman A. (1980). Patients and Healers in the Context of Culture: An Exploration of the Borderland between Anthropology, Medicine, and Psychiatry.

[51] Modjadji P. (2019). Growth Patterns and Socio-Cultural Beliefs and Practices in Dikgale, Limpopo Province: A Mixed Method Study of Primary School Children and Their Mothers. Ph.D. Thesis.

[52] Leroy J.L., Ruel M., Habicht J.-P., Frongillo E.A. (2014). Linear growth deficit continues to accumulate beyond the first 1000 days in low-and middle-income countries: Global evidence from 51 national surveys. J. Nutr..

[53] Tong A., Sainsbury P., Craig J. (2007). Consolidated criteria for reporting qualitative research (COREQ): A 32-item checklist for interviews and focus groups. Int. J. Qual. Health Care.

[54] Young H., Marshak A. (2017). Persistent Global Acute Malnutrition.

[55] Rahman M. (2017). The advantages and disadvantages of using qualitative and quantitative approaches and methods in language “testing and assessment” research: A literature review. J. Educ. Learn..

[56] Modjadji P., Madiba S. (2019). The double burden of malnutrition in a rural health and demographic surveillance system site in South Africa: A study of primary schoolchildren and their mothers. BMC Public Health.

[57] Modjadji P., Madiba S. (2019). Childhood undernutrition and its predictors in a rural health and demographic surveillance system site in South Africa. Int. J. Environ. Res. Public Health.

[58] Modjadji P. (2020). Socio-demographic determinants of overweight and obesity among mothers of primary school children living in a rural health and demographic surveillance system site, South Africa. Open J. Public Health.

[59] Alberts M., Dikotope S.A., Choma S.R., Masemola M.L., Modjadji S.E., Mashinya F., Burger S., Cook I., Brits S.J., Byass P. (2015). Health & demographic surveillance system profile: The Dikgale health and demographic surveillance system. Int. J. Epidemiol..

[60] Damen F.W.M., Luning P.A., Fogliano V., Steenbekkers B.L.P.A. (2019). What influences mothers’ snack choices for their children aged 2–7?. Food Qual. Prefer..

[61] Gauthier A.H., Smeeding T.M., Furstenberg F.F.J. (2004). Are parents investing less time in children? Trends in selected industrialized countries. Popul. Dev. Rev..

[62] Zhang P., Wu J., Xun N. (2019). Role of maternal nutrition in the health outcomes of mothers and their children: A retrospective analysis. Med Sci. Monit..

[63] Hsieh H.-F., Shannon S.E. (2005). Three approaches to qualitative content analysis. Qual. Health Res..

[64] Smith J.A., Flowers P., Larkin M. (2009). Interpretative Phenomenological Analysis: Theory, Method and Research.

[65] Nkurunziza S., Meessen B., Van geertruyden J.-P., Korachais C. (2017). Determinants of stunting and severe stunting among Burundian children aged 6–23 months: Evidence from a national cross-sectional household survey. BMC Pediatrics.

[66] Mosli R.H., Miller A.L., Peterson K.E., Lumeng J.C. (2016). Sibling feeding behavior: Mothers as role models during mealtimes. Appetite.

[67] Palfreyman Z., Haycraft E., Meyer C. (2015). Parental modelling of eating behaviours: Observational validation of the Parental Modelling of Eating Behaviours scale (PARM). Appetite.

[68] Pesch M.H., Appugliese D.P., Kaciroti N., Rosenblum K.L., Miller A.L., Lumeng J.C. (2016). Maternal encouragement and discouragement: Differences by food type and child weight status. Appetite.

[69] Modjadji P., Mashishi J. (2020). Persistent malnutrition and associated factors among children under five years attending primary health care facilities in Limpopo Province, South Africa. Int. J. Environ. Res. Public Health.

[70] Schrempft S., Van Jaarsveld C.H., Fisher A., Fildes A., Wardle J. (2016). Maternal characteristics associated with the obesogenic quality of the home environment in early childhood. Appetite.

[71] Habibi M., Laamiri F.Z., Aguenaou H., Doukkali L., Mrabet M., Barkat A. (2018). The impact of maternal socio-demographic characteristics on breastfeeding knowledge and practices: An experience from Casablanca, Morocco. Int. J. Pediatr. Adolesc. Med..

[72] Galgamuwa L.S., Iddawela D., Dharmaratne S.D., Galgamuwa G. (2017). Nutritional status and correlated socio-economic factors among preschool and school children in plantation communities, Sri Lanka. BMC Public Health.

[73] Seifu C.N., Whiting S.J., Henry C.J., Belachew T., Hailemariam T.G. (2015). Association between maternal and child nutritional status in Hula, rural southern Ethiopia: A cross sectional study. PLoS ONE.

[74] Bilal S.M., Moser A., Blanco R., Spigt M., Dinant G.J. (2014). Practices and challenges of growth monitoring and promotion in Ethiopia: A qualitative study. J. Health Popul. Nutr..

[75] Mangasaryan N., Arabi M., Schultink W. (2011). Revisiting the concept of growth monitoring and its possible role in community-based nutrition programs. Food Nutr. Bull..

[76] Robert R.C., Gittelsohn J., Creed-Kanashiro H.M., Penny M.E., Caulfield L.E., Narro M.R., Black R.E. (2006). Process evaluation determines the pathway of success for a health center-delivered, nutrition education intervention for infants in Trujillo, Peru. J. Nutr..

[77] Dekker L.H., Mora-Plazas M., Marín C., Baylin A., Villamor E. (2010). Stunting associated with poor socioeconomic and maternal nutrition status and respiratory morbidity in Colombian schoolchildren. Food Nutr. Bull..

[78] Hossain M., Ickes S., Rice L., Ritter G., Naila N.N., Zia T., Nahar B., Mahfuz M., Denno D.M., Ahmed T. (2018). Caregiver perceptions of children’s linear growth in Bangladesh: A qualitative analysis. Public Health Nutr..

[79] Roberfroid D., Pelto G.H., Kolsteren P. (2007). Plot and see! Maternal comprehension of growth charts worldwide. Trop. Med. Int. Health.

[80] Flaherty-Hewitt M. (2016). Nutrition and Growth Measurement. http://wwwemedicinemedscapecom/article/1948024-overview.

[81] Ventura A.K., Worobey J. (2013). Early influences on the development of food preferences. Curr. Biol..

[82] Birch L.L. (1999). Development of food preferences. Annu. Rev. Nutr..

[83] Russell C.G., Worsley A. (2013). Why don’t they like that? And can I do anything about it? The nature and correlates of parents’ attributions and self-efficacy beliefs about preschool children’s food preferences. Appetite.

[84] Fink S.K., Racine E.F., Mueffelmann R.E., Dean M.N., Herman-Smith R. (2014). Family meals and diet quality among children and adolescents in North Carolina. J. Nutr. Educ. Behav..

[85] Jaworowska A., Blackham T., Davies I.G., Stevenson L. (2013). Nutritional challenges and health implications of takeaway and fast food. Nutr. Rev..

[86] Suglia S.F., Shelton R.C., Hsiao A., Wang Y.C., Rundle A., Link B.G. (2016). Why the neighborhood social environment is critical in obesity prevention. J. Hered..

[87] Graham L., Hochfeld T., Stuart L., Van Gent M. (2015). Evaluation Study of the National School Nutrition Programme and the Tiger Brands Foundation in School Breakfast Feeding Programme in the Lady Frere and Qumbu Districts of the Eastern Cape.

[88] HAKSA Report Card. https://www.ssisa.com/files/wp-content/uploads/2018/11/HAKSA-2018-Report-Card-FINAL.pdf.

[89] Ekesa B., Blomme G., Garming H. (2011). Dietary diversity and nutritional status of pre-school children from Musa-dependent households in Gitega (Burundi) and Butembo (Democratic Republic of Congo). Afr. J. Food Agric. Nutr. Dev..

[90] Ogechi U.P., Chilezie O.V. (2017). Assessment of dietary diversity score, nutritional status and socio-demographic characteristics of under-5 children in some rural areas of Imo State, Nigeria. Malays. J. Nutr..

[91] Modjadji P., Molokwane D., Ukegbu P.O. (2020). Dietary diversity and nutritional status of preschool children in North West Province, South Africa: A cross sectional study. Children.

[92] Bella H., Al-Ansari S.S. (1999). Factors affecting child development in Madinah, Saudi Arabia. J. Fam. Community Med..

[93] Rogol A.D. (2016). Growth and maturation of children and adolescents. Oxf. Handb. Econ. Hum. Biol..

[94] Kelly G.E., Murrin C., Viljoen K., O’Brien J., Kelleher C. (2014). Body mass index is associated with the maternal lines but height is heritable across family lines in the Lifeways Cross-Generation Cohort Study. BMJ Open.

[95] Baron J., Sävendahl L., De Luca F., Dauber A., Phillip M., Wit J.M., Nilsson O. (2015). Short and tall stature: A new paradigm emerges. Nat. Rev. Endocrinol..

[96] Aitsi-Selmi A. (2014). Households with a stunted child and obese mother: Trends and child feeding practices in a middle-income country, 1992–2008. Matern. Child Health J..

[97] De Onis M., Branca F. (2016). Childhood stunting: A global perspective. Matern. Child Nutr..

[98] Goudet S.M., Faiz S., Bogin B.A., Griffiths P.L. (2011). Pregnant women’s and community health workers’ perceptions of root causes of malnutrition among infants and young children in the slums of Dhaka, Bangladesh. Am. J. Public Health.

