# Supplementary material for: Engaging Mothers on the Growth of School-Age Children in a Rural South African Health and Demographic Site: A Qualitative Insight
Source: Healthcare (Basel). 2021 Feb 18;9(2):225. doi: 10.3390/healthcare9020225 (PMC7922540; doi:10.3390/healthcare9020225)
Supplement: Supplementary file 1 [file healthcare-09-00225-s001.pdf]

healthcare

Greetings to the participants

- Introduction of the study and the observer by the researcher,
- Participants are given opportunity to ask questions,
- Administration of the consent forms
- Administration of sociodemographic information sheet
- And, putting pseudonyms in preparation for the focus group discussion

Explanation of purpose

”Thank you for agreeing to participate in this focus group discussion. The purpose of today’s discussion is to explore your insights on child growth as mothers with regard to your child who participated in the study with you, initially. We want to know how you view child growth in general, and whether your child is growing well. You are experienced mothers and you continue to feed your children. We would appreciate your open mind during the focus group discussion. Feel free to raise a point that you consider important. Also, please feel free to tell me when you are not comfortable about any of the questions, but I would appreciate if you could answer as many of the questions as possible.”

Rules of engagement are explained and most importantly, participants are reminded that the discussion are confidential.

**Questions and probes**	
1.	What is your understanding about child growth?<br>**Probes**<br>• What do you think lead to children not to grow in the same way.<br>• What are the things you think affect child growth?
2.	In your opinion, how does food affect child growth?<br>**Probes**<br>• Which foods are important for child growth?<br>• Which foods are not good to be eaten by a child?
3.	In your views, do you think your children have adequate food on daily basis at home or at school?<br>**Probes**<br>• When you say a child eats adequate how do you come to that?<br>• When you say a child does not eat adequate how do you come to that?<br>• When you say a child does not eat adequate what are the reasons?<br>• Who decides which foods to purchase?
4.	In your community what are some of the things or beliefs that influence which foods to eat?<br>**Probe for**:<br>• Cultural beliefs and practices in community pertaining to child nutrition.<br>• Religious beliefs pertaining to child nutrition.<br>• Foods which are not supposed to be given to children because of culture.
5.	During pregnancy, what are some of the foods you were not supposed to eat because they are culturally forbidden?<br>**Probe for**<br>• Religious beliefs pertaining to child nutrition.
6.	During pregnancy, what are some of the things that you were eating which you usually do not eat?<br>**Probe** for soil, ashes, red clay rock etc. and why?
7.	What are your views on the way culture prescribe:<br>• For some of the foods that we should or should not eat, give, or not give our children?<br>• On Child growth?

This is the end of our focus group discussion.

Thank you very much for your time and insights.

We appreciate all that you had to say.
